# Modes of Leaflet Fluttering: Quantitative Characterization of a Bovine Bioprosthetic Heart Valve

**DOI:** 10.1007/s10439-025-03906-9

**Published:** 2025-11-14

**Authors:** Silje Ekroll Jahren, Bernhard Vennemann, Karoline-Marie Bornemann, Thomas Rösgen, Dominik Obrist

**Affiliations:** 1 ARTORG Center for Biomedical Engineering Research, University of Bern, Freiburgstrasse 3, 3010 Bern, Switzerland; 2 Institute of Fluid Dynamics, ETH Zürich, Zurich, Switzerland; 3 Department of Pediatrics (Cardiology), Stanford University, Stanford, CA USA

**Keywords:** Bovine bioprosthetic heart valves, Leaflet fluttering, Structural valve deterioration, Fluid-structure interaction

## Abstract

Oscillations of aortic bioprosthetic heart valve (BHV) leaflets during systole are known as leaflet fluttering (LF). LF may be relevant for assessing valvular function and could play a role in structural valve deterioration. However, a quantitative characterization of LF and its underlying physical processes is still missing. The objectives of this study are to systematically characterize LF for a BHV in vitro and to investigate the associated flow structures using a computational model. Leaflet motion of a bovine BHV was captured with high-speed cameras in an in vitro flow loop under varying experimental conditions (cardiac output (CO), inflow, and valve and aortic root orientation). A fluid-structure interaction (FSI) study was conducted for the same BHV for one condition to examine the blood flow patterns associated with LF. In vitro, LF presents in two different modes: either as high-frequency (150–380 Hz), low-amplitude (0.2–0.8 mm) vibrations of the leaflet tips (V-mode) or low frequency (30–90 Hz), high-amplitude (0.4–2.6 mm) waves travelling from leaflet base to tip (T-mode). We observed that LF depends on individual leaflet properties, is more likely to occur, and increases in amplitude and frequency with higher CO, and is also affected by the inflow. The FSI study confirms the presence of the same two modes. We identified large-scale vortex shedding related to the T-mode, superimposed with small-scale vortex shedding connected to vibrations of the leaflet tip (V-mode). Both identified LF modes are potential factors in BHV degeneration and should be considered in the BHV’s design.

**Supplementary Information:**

The online version contains supplementary material available at 10.1007/s10439-025-03906-9.

## Introduction

Bioprosthetic heart valves (BHVs) are believed to offer better hemodynamics and lower thrombogenic potential compared to mechanical heart valves [1]. The success of transcatheter aortic valve implantation (TAVI), together with increasing numbers of younger patients who refuse lifelong anticoagulation therapy, has increased the number of patients who receive BHVs [2]. However, BHVs are prone to structural valve deterioration (SVD) such as pannus formation, calcification and fibrosis of the leaflets [1, 3], delamination, and perforations and ruptures [1]. The mechanisms underlying SVD are not yet fully understood. However, SVD is a long-term immune rejection and tissue remodeling process [1], and is partially a result of a mechanical fatigue process [4, 5]. Vibrations of valve leaflets during the systolic phase, known as leaflet fluttering (LF), could contribute to this fatigue process and have been associated with parameters linked to calcification and thrombosis [6].

In healthy native aortic valves, reports of LF are rare. It was proposed in the 1980 s as a marker to exclude aortic stenosis [7]. Diastolic LF has been reported for aortic leaflet flail [8]. Rameh and Kossaify [9] observed aortic LF mainly in the non-coronary leaflet, and minimal fluttering was also observed for the right coronary leaflet. They concluded that systolic LF is rare and might occur as a primary or secondary dysfunction, and that the physiopathology of LF is poorly understood.

LF in BHVs has received more attention. Clinically, LF has been reported in stentless BHVs [10], and a vibratory mode or “hum” has been observed in transcatheter aortic valve implantion (TAVI) patients [11]. The lack of “humming” was suggested as an indicator for impaired leaflet motion possibly caused by valve thrombosis. In the 1990 s low-frequency LF (15-30 Hz) was observed in vitro for BHVs using water as circulating fluid [12], and LF was linked to the development of a turbulent sinus vortex. Moore and Dasi [13] reported high frequency (200 Hz, 0.5 mm amplitude) and low frequency (50–100 Hz, 4 mm) fluttering during systole for a porcine BHV and saline water. In their computational study, Becsek, Pietrasanta and Obrist [14] reported LF at 40 Hz, which agreed well with the frequency of 38 Hz measured experimentally by Vennemann et al. [15]. Becsek et al. [14] characterized LF kinematics as a whiplash-like motion of the leaflets resulting in a travelling wave from the base to the tip of the leaflets with a wave speed of 0.4 m/s. They also connected LF to the periodic shedding of vortex rings at 40 Hz from the valve orifice during systolic ejection. The whiplash-like LF kinematics were confirmed in the computational study of Fringand et al. [16].

LF has been linked to leaflet stiffness [12, 17] and elastic modulus, leaflet thickness[18, 19], sinus size [20], tissue type [15, 17, 20, 21], and viscosity of the fluid [12, 13]. Corso and Obrist [22] showed that the amplitude and character of LF depend on the leaflet geometry. Avelar et al. [23] found that high-frequency LF increased in frequency and amplitude for higher cardiac output (CO) and argued that fluttering might cause regurgitation and accelerate calcification and fatigue. The potential connection between LF and calcification was also highlighted by Tsolaki et al. [24]. Bornemann and Obrist [25] linked LF to a fluid-structure instability triggered by a shear layer instability downstream of the valve orifice. Most recently, Bornemann and Obrist [26] showed that the character of the laminar-turbulent transition of the systolic jet is strongly affected by the presence of LF. These studies suggest that controlling LF through appropriate BHV design could be an effective means to modulate turbulent blood flow downstream of BHVs, potentially even helping to mitigate SVD.

Nevertheless, only a few studies exist that focus primarily on LF [6, 17, 19, 23, 27–29]. Moreover, some of the reported results are conflicting: In some studies [12, 13] viscosity-matched fluid (glycerin-mixture) eliminates LF, while other studies (e.g., Vennemann et al. [15]) report LF also for such fluid. In some studies, LF is enhanced for lower cardiac output [20] while in other LF is enhanced for higher cardiac output [23]. Reported fluttering frequencies range from 15 to 750 Hz with amplitudes reported up to 4 mm. It remains unclear whether these differences indicate the existence of different fluttering modes, whether the differences are due to different flow conditions, different valve designs, different aortic root morphology, or different experimental setups. To help clarify some of these questions, we aim here to a) characterize LF for a specific BHV in vitro by systematically changing the experimental configuration (e.g., CO and valve orientation) and b) to identify flow structures associated with LF by performing numerical simulations with the same BHV geometry.

## Materials and Methods

### In Vitro Experiments

#### Experimental Setup

**Fig. 1:**
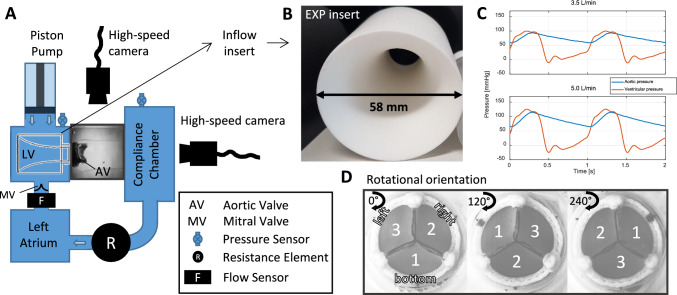
**A** Schematic of the left heart flow loop. **B** The exponential horn (EXP) insert used to modify the inflow to the BHV. **C** Pressure waveforms during the two different hemodynamic configurations (1 and 2) for the left ventricular pressure and the aortic pressure measured in the compliance chamber. **D** The three different rotational orientations of the phantom and valve: leaflet 1 and sinus 1 are in the bottom position (left), leaflet 2 and sinus 2 are in the bottom position (middle), and leaflet 3 and sinus 3 are pointing in the bottom position (right).

A surgical BHV (Edwards Intuity Elite 21 Valve, Edwards Lifesciences, Irvine, CA, USA) was mounted in an aortic root phantom with an annulus diameter of 21 mm. The phantom approximated the anatomy of the human aortic root and comprised three sinus portions of equal size [30, 31]. It was fabricated from transparent silicone (ELASTOSIL RT 601 A/B silicone, Wacker Silicones, Wacker Chemistry AG, Munich, Germany, properties: 1.02 g/cm^3^ density, 45 hardness Shore A, 6 N/mm^2^ tensile strength, 100% elongation at break, 2.8 permittivity), in a layer-by-layer approach to a negative model (see Supplementary Figure 1), to provide full optical access and physiological distensibility (approx. 0.3%/mmHg) [32, 33]. The phantom was immersed in a fluid-filled chamber (using same fluid as the circulating fluid) and inserted into a pulsatile left heart flow loop (Figure 1A) as described in Jahren et al. [32]. The circulating fluid was a mixture of glycerin (34% by weight), sodium chloride (16.6%), and water (49.4%), which was index-matched to the silicone of the aortic root phantom to allow for undistorted optical access. The kinematic viscosity of the fluid was $4.3\bullet {10}^{-6}$ m^2^/s, which is higher, but close to the viscosity of blood ($2.8 - 3.8 \times {10}^{-6}$ m^2^/s).

#### Instrumentation

A flow sensor (TS410/ME-11PXL, Transonic Systems Inc., Ithaca, NY, USA) and two pressure sensors (CODAN xtrans, CODAN pvb Critical Care GmbH, Forstinning, Germany) positioned in the model of the left ventricle and the compliance chamber were used to tune the flow loop to replicate physiological flow and pressure profiles. Two high-speed cameras (FASTCAM Mini AX100, Photron USA Inc., San Diego, CA, USA) were positioned perpendicularly and axially to the aortic root phantom to record images of the valve from radial and axial view, respectively. All measurements were synchronized using a trigger signal from the piston pump (ViVitro Systems Inc. Victoria, Canada), which actuated the flow through the BHV. The trigger signal was activated using a switch after the hemodynamic configuration was set and stabilized. The pressure and flow measurements were performed at 200 Hz for 15 s, and the high-speed camera videos were recorded at 2000 frames/second for the first 1.5 s after the trigger.

#### Measurement Protocol

The measurement protocol was designed to systematically test the effect of the following factors on leaflet fluttering: (i) hemodynamics (flow rate and pressure), (ii) inflow to the valve, (iii) valve asymmetry (leaflet differences), and (iv) aortic root phantom asymmetry (imperfections).

Figure 1 shows a schematic of the in vitro flow loop and the different experimental configurations. To study the impact of (i) hemodynamics on LF, two different hemodynamic configurations were tested at a heart rate of 60 bpm [32, 34]:

1. Cardiac output of 3.5 l/min, aortic pressure of 100/60 mmHg
2. Cardiac output of 5.0 l/min, aortic pressure of 120/70 mmHg

Figure 1C shows an example of the pressure waveforms for the two different hemodynamic configurations. The impact of the (ii) inflow to the BHV was tested by changing the geometric configuration of the left ventricular chamber by using an exponential horn (EXP) insert in the left ventricular outflow tract (Figures 1A, B, Supplementary Figure 2). To test for the effect of (iii) experimental asymmetries in the valve configuration, e.g., gravitational forces, asymmetric inflow to the test section, and valve asymmetry three different rotational orientations of the BHV and aortic root with respect to the flow loop were tested (Figure 1 1D). The impact of (iv) possible imperfections of the aortic root phantom was investigated by rotating the valve within the phantom, such that each leaflet was tested in each sinus portion of the phantom. Table 1 shows a detailed overview of the experimental protocol.

**Table 1:** Overview of the measurement protocol

Measurement	Hemodynamics		Inflow LVOT	Valve orientation	Root orientation
Number	Cardiac output	Aortic pressure systolic/diastolic	Type of insert	Leaflet at bottom	Sinus at bottom
[-]	[L/min]	[mmHg]	[–]	[–]	[–]
1	3.5	100/60	NO	1	1
2				2	2
3				3	3
4	5.0	120/70	NO	1	1
5				2	2
6				3	3
7	3.5	100/60	EXP	1	1
8				2	2
9				3	3
10	3.5	100/60	EXP	2	1
11				3	1
12	5.0	120/70	EXP	1	1
13				2	2
14				3	3
15	5.0	120/70	EXP	2	1
16				3	1

Inflow, valve, and root orientations are defined in Figure 1

*LVOT* left ventricular outflow tract, *NO* no insert, *EXP* exponential horn

#### Data Analysis

**Fig. 2:**
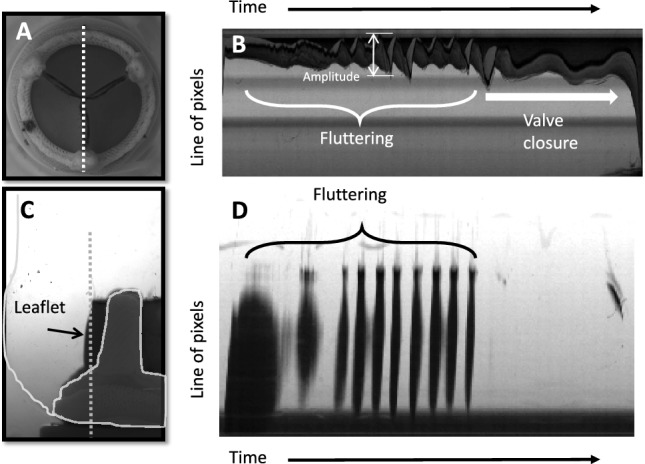
Post-processing of the images: **A** axial view of BHV with the dotted line indicating the line of pixels stacked over time in **B**. **B** Stacked image of the pixel lines indicated in **A** showing fluttering of the top leaflet tip. **C** Radial view of BHV with the dotted line indicating the line of pixels stacked over time. **D** Stacked image of the pixel lines indicated in **C** showing waves traveling through the leaflet from the base to the leaflet tip.

The recorded images were digitally post-processed. In Vennemann et al. [15], we showed that the leaflet motion is strongest in the middle of the free edge of leaflets. Therefore, we focused our analysis on this region. Leaflet tip motion was studied by stacking (frame by frame) lines of pixels in radial direction from the valve center to the stent ring (axial view) during systole (Figure 2A, B). Fluttering frequency and amplitude were determined from the axial view stacks of pixels (Figure 2B). The leaflet motion from base to tip was investigated by stacking lines of pixels in axial direction along the edge of the valve leaflet (radial view, Figure 2C, D). From the radial view stacks the wave motion along the leaflet from base to tip was studied (Figure 2D). The fluttering frequencies of the tip were determined using a Fourier transform over the whole systolic time to identify the dominant frequencies of the time series. If the fluttering showed fewer than five consecutive fluttering periods, it was excluded from the analysis. From these frequencies, only the maximum frequency is reported. The fluttering amplitudes were determined as the distance between the outermost and innermost tip positions of the leaflet for all vibrations per heartbeat. Multiple analysis of variance (ANOVA) was used to test for statistically significant differences across the different experimental configurations with respect to fluttering parameters, e.g., frequency and amplitude. If the ANOVA indicated significance (p < 0.05), pairwise t-tests were performed to determine which experimental configurations were significantly different from others.

### Computational Model

**Fig. 3:**
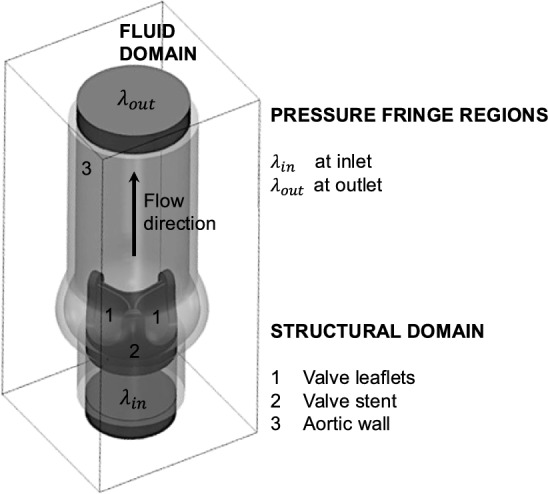
Computational setup: Structural domain, including the valve leaflets (1), the valve stent (2), and the aortic wall (3) immersed in the fluid domain (enclosing box). The fringe regions were positioned at the inlet (${\lambda }_{in})$ and outlet (${\lambda }_{out}$) to impose a transvalvular pressure gradient over the valve.

A computational simulation was conducted to assess the FSI connected to the experimentally observed LF, to investigate the driving mechanisms of the fluttering, and to exclude the possibility that LF is an experimental artifact. Figure 3 shows the computational model setup, including the structural and fluid domains. A fiber-reinforced BHV model was created based on the BHV used in the presented in vitro experiments. The valve was inserted into a generic aortic root geometry with dimensions consistent with those of the experimental aortic root phantom.

The interaction between blood flow and flexible, hyperelastic leaflet tissue was modeled within a high-fidelity FSI solver [35]. A high-order Navier-Stokes solver, using a Direct Numerical Simulation approach [36], computed the flow field using sixth-order finite differences on a structured, staggered grid of 193 x 193 x 513 elements. This resulted in a minimal mesh width of 125 µm in the immediate leaflet vicinity. A finite element method was used for solving the elastodynamic equations on the structural mesh of approximately 450,000 tetrahedral elements. Fluid and structure were coupled to the fluid solver by an Immersed Boundary Method where the fluid velocities and the structural forces were exchanged between the fluid grid and finite element mesh based on variational transfer. Boundary conditions were imposed using the fringe region technique resulting in an average systolic transvalvular pressure gradient of $8$ mmHg (see Figure 3 for the position of the fringe regions). The time step is set to $\Delta t= 2.5\cdot {10}^{-6}$ s. Details on numerical schemes, solver performance and numerical validation can be found in Nestola et al. [35].

Aortic wall and valve stent were assigned linear-elastic material properties. A fiber-reinforced Holzapfel-Gasser-Ogden model was applied for the biological leaflet tissue with material parameters consistent with the choice of Auricchio et al. [37] for bovine pericardium (µs = 20.1 kPa, k11 = k12 = 54.62 kPa, k21 = k22 = 30.86, ρs = 1, 100 kg/m3). Two sets of fibers were placed in each leaflet. The fiber orientation was set to 30° relative to the middle plane of each leaflet, resulting in a relative angle of 60° between the two fiber sets. The fluid was assigned Newtonian and incompressible behavior, material properties were chosen similar to blood (dynamic viscosity ${\mu }_{f}=0.004\text{Pa s}$, density ${\rho }_{f}=1050\mathrm{kg/}{\mathrm{m}}^{3}$). A transvalvular pressure gradient across the valve prosthesis was imposed and tuned to mimic the increasing flow rate during systolic acceleration. A cardiac output of 5.0l/min was prescribed. A validation of the resulting flow field against experimental results is presented in [14].

## Results

### In Vitro Results

#### Fluttering Modes

**Fig. 4:**
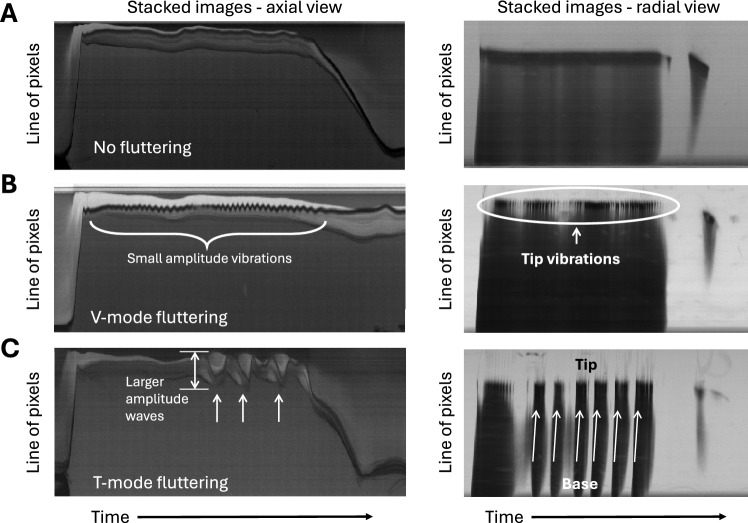
Examples of different fluttering modes seen from axial view (left) visualizing leaflet tip displacement and from radial view (right) visualizing leaflet displacement from base to tip. **A** No fluttering, **B** V-mode fluttering: high-frequency leaflet tip vibration with small amplitude. **C** T-mode fluttering: waves travelling through the leaflet with larger amplitudes. In **C** radial view V- and T-mode fluttering can be seen in the same leaflet.

In the 16 experiments (Table 1) four different types of leaflet motion were observed during peak systole: Either no LF (Figure 4A), LF with high-frequency tip vibrations and low amplitude (Figure 4B), low-frequency LF with larger amplitude waves travelling from leaflet base to tip (Figure 4C) or a combination of tip vibrations and travelling waves (Figure 4C, radial view). These observations suggest the definition of two fluttering modes: *V-mode fluttering,* referring to high-frequency tip vibrations, and *T-mode fluttering,* referring to low-frequency travelling waves. An animated illustration for V-mode and T-mode fluttering is provided in Supplementary Video 1. Table 2 gives an overview of typical leaflet fluttering frequencies, amplitudes, and occurrence of the two different modes of fluttering.

**Table 2:** Leaflet fluttering frequency, amplitude, and occurrence for the two different observed fluttering modes

Fluttering mode	Frequency	Amplitude	Occurrence
[–]	[Hz]	[mm]	[–]
V	150-380	0.2-0.6	Continuous
T	30-90	0.8-2.3	Single events (1–7 waves)

*V* leaflet tip vibrations, *T* travelling waves from leaflet base to tip

**Fig. 5:**
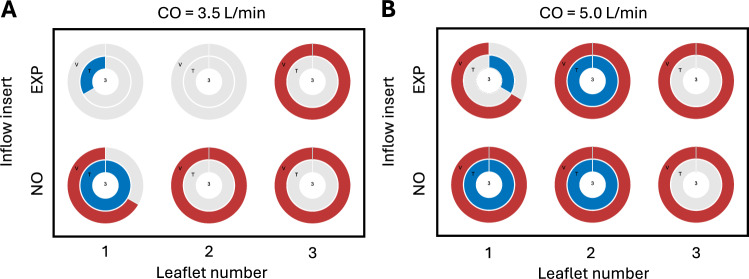
Occurrence of V-mode (red) and T-mode (blue) for different experimental configurations. The central number in each diagram indicates the number of measurements. Simultaneous occurrence of V- and T-mode is indicated by overlapping rings. Occurrence for the different inflow inserts (no insert = NO, exponential flow insert = EXP) and different leaflets (1,2,3) for **A** CO of 3.5 l/min and **B** 5.0 l/min, respectively (combinging results from 3 different valve orientations).

The composite diagrams in Figure 5A and B report the occurrence of V-mode and T-mode fluttering with respect to inflow (NO and EXP insert) and leaflet number for different CO, respectively. In general, there was more fluttering for higher CO. For CO = 3.5 l/min, the EXP insert reduced the occurrence of LF (both modes) for leaflets 1 and 2. For CO = 5.0 l/min, the effect of the EXP insert was weaker and only evident for leaflet 1.

All data in Figure 5 indicate a strong dependency of T-mode fluttering on the individual leaflet: T-mode never occurred on leaflet 3, but frequently on leaflet 1 and leaflet 2. In contrast, leaflet 3 exhibited V-mode fluttering in all measurements. An overview of all measurement data including fluttering modes, frequencies, amplitudes, and occurrences, are provided in Supplementary Table 1.

#### V-Mode Fluttering

V-mode fluttering (tip vibrations, Figure 4B) was typically continuous with one dominant frequency and amplitude (Table 2). In some cases, this fluttering did not persist for five or more consecutive periods and was therefore excluded. Supplementary Figure 3A, B, E and F show characteristic V-mode fluttering.

**Fig. 6:**
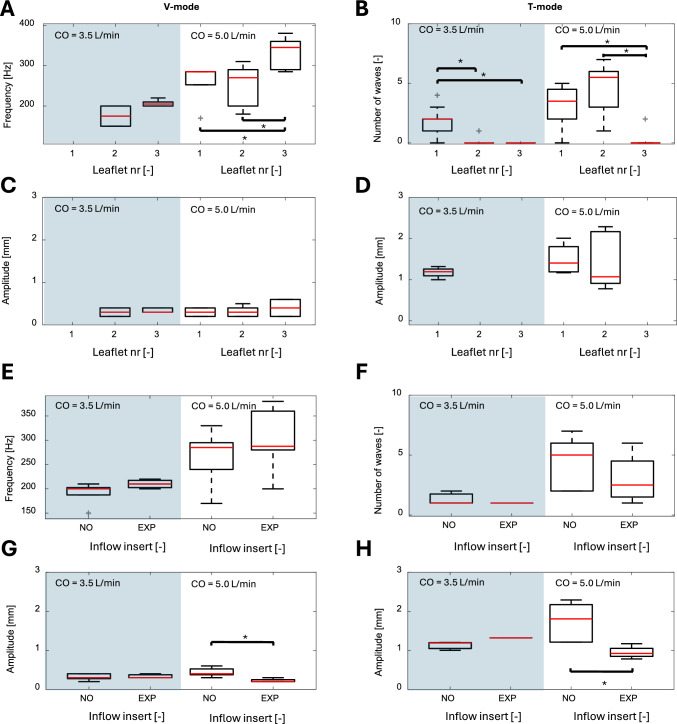
Frequency, amplitude, and wave occurrence of V- and T-mode fluttering for different cardiac output (CO) and inflow inserts. Grey area (left) marks measurements with CO = 3.5 L/min and white area (right) marks measurements with CO = 5.0 L/min in each plot. **A** Boxplots of the maximal frequency and **C** maximal amplitude observed for V-mode for all leaflets (1-3). **B** Boxplots of the number of waves and **D** maximal amplitude observed for T-mode for all leaflets. **E** Boxplots of the maximal frequency and **G** maximal amplitude observed for V-mode for the two different inflow inserts (NO and EXP). **F** Boxplots of the number of waves and **H** the maximal amplitude observed for T-mode for the two different inflow inserts and for two different CO. The red lines represent the median values, the grey crosses (+) indicate outliers, and the black stars (*) indicate significant differences in means between the groups connected with the black solid lines.

Figure 6A and C show the influence of CO and individual leaflet properties on LF frequency and amplitude of the V-mode. The frequency increased significantly for higher CO (p = 0.0006), and leaflet 3 exhibited a significantly higher frequency than leaflets 1 and 2 for CO = 5.0 l/min (p = 0.0198 and p = 0.0002, respectively). The V-mode fluttering amplitude did not depend on the CO (p = 0.7645), but it tended to be higher on leaflet 3 compared to leaflet 1 and 2 for CO = 5.0 l/min. Figure 6E and G show the influence of CO and inflow insert on LF frequency and amplitude of the V-mode. The V-mode frequency was found to be independent of the inflow (p = 0.1323). In contrast, the amplitude was significantly lower for EXP compared to NO for CO = 5.0 l/min (p = 0.0002) and for all measurements together (p = 0.0014). The rotational orientation of the BHV did neither significantly affect the frequency nor the amplitude.

#### T-Mode Fluttering

T-mode fluttering (travelling waves from base to tip, Figure 4C) was characterized by a slow retraction of the leaflet followed by a fast wave-like whiplash motion (Supplementary Figure 3C, D, G, and Supplementary Video 1). The phase speed of these travelling waves can be estimated from their slope in the radial view stacks. This yielded velocities in the range of 0.7-1.4 m/s, with an uncertainty of approximately ± 0.5 m/s, due to limited spatial and temporal resolution.

In contrast to V-mode fluttering, the T-mode occurred intermittently during the systolic phase, and it was difficult to assign a specific fluttering frequency. Therefore, the number of waves occurring during a single heartbeat is reported instead. If interpreted as vibration, frequencies in the range of 30-90 Hz were observed. Figure 6B and D show the influence of CO and individual leaflet properties on the number of waves and amplitude of the T-mode. The number of waves, ranging from zero to seven, increased significantly for higher CO (p = 0.0333), and depended significantly on the individual leaflet (p = 0.0378). The T-mode amplitude, ranging from 0.8 to 2.3 mm, tended to increase for higher CO, but did not depend on the individual leaflet (p = 0.8725). Figure 6F and H show the influence of CO and inflow insert on the number of waves and amplitude of the T-mode. The T-mode number of waves was found to be statistically independent of the inflow (p = 0.5092), while the amplitude was found to be significantly lower for EXP compared to NO for CO = 5.0 l/min (p = 0.0242), and for all measurements together (p = 0.0274). Finally, the rotational orientation of the BHV did not influence the number of waves or the amplitude.

### Computational Results

**Fig. 7:**
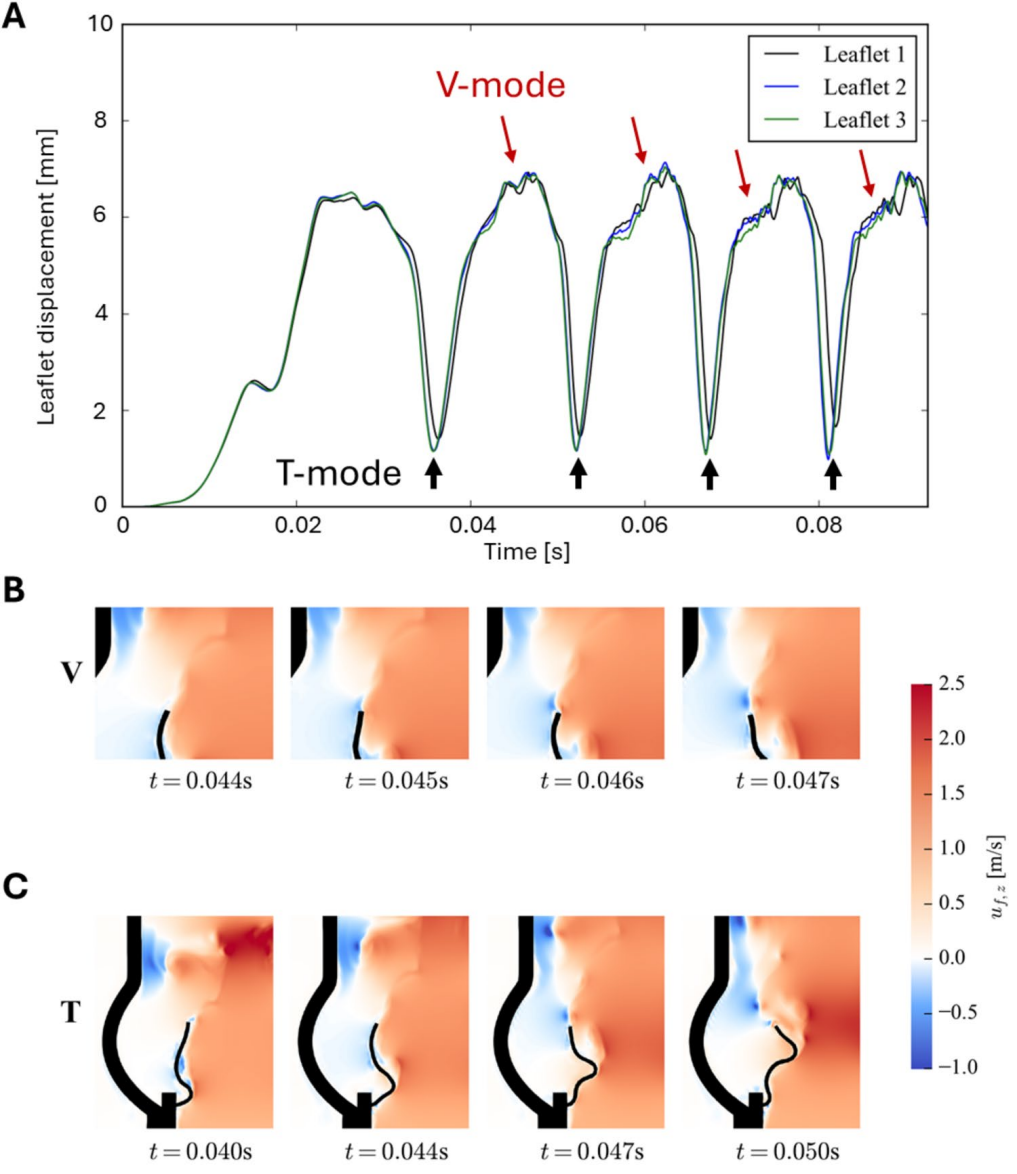
**A** Leaflet displacements for each leaflet over time. **B** Streamwise velocity field around one valve leaflet with V-mode from t = 0.044 s to 0.047 s and **C** T-mode from t = 0.040s to 0.050s.

The computational model simulated the flow and the valve kinematics during peak systole. After an initial transient, the valve leaflets exhibited sustained LF. Figure 7A shows the displacement of each individual leaflet over time. In accordance with the experimental results, the numerical simulation reveals vibrations of the leaflet tip (V-mode), traveling wave motion of the entire leaflet (T-mode), and asynchronous behavior of the leaflets. For V-mode, we see a dominant frequency of approximately 770 Hz and an amplitude of 0.4 mm. However, multiple frequencies seem to be superimposed. For the T-mode, both frequency and amplitude differ significantly from those of the V-mode, with a frequency of 67 Hz and an amplitude of 5.5 mm. The leaflets exhibit asynchronous behavior, with one leaflet having a somewhat lower amplitude of the T-mode and a later onset of the V-mode compared to the other two leaflets. Figure 7B and C show the streamwise velocity field around one leaflet for V-mode and T-mode LF, respectively. V-mode only affects the leaflet tip and is connected to the shedding of small vortices, which are then advected downstream. V-mode is only observed when the leaflet is in its most open position. T-mode affects the entire leaflet leading to large displacements. During the initial leaflet opening (t = 0.04 s), a recirculation area is created within the S-shaped leaflet. As the travelling wave progresses through the leaflet starting from the attachment point, fluid is pushed downstream, and we observe an acceleration of the flow at the sharp leaflet curve (t = 0.044 s). When this curve moves downstream, a vortex forms, which appears to be created by flow separation at the sharp leaflet curve (t = 0.047 s). In the final step of one T-mode fluttering cycle (t = 0.05 s), the travelling wave reaches the leaflet tip leading to a large displacement. During this movement, fluid is pushed out rapidly leading to the shedding of a large vortex ring (as also reported in Bornemann and Obrist, 2025).

## Discussion

In this observational study, we aimed to systematically quantify leaflet fluttering (LF) in BHVs and to study the effect of different experimental settings on the LF characteristics (e.g., amplitude and frequency). We identified two fundamentally different LF modes: 1) V-mode with continuous high-frequency, low amplitude tip vibrations, and 2) T-mode with low frequency or single event, high amplitude waves travelling from leaflet base to leaflet tip (Table 2). We made three basic observations valid for both fluttering modes: LF: (i) is more likely for higher flow rates, (ii) is affected by the characteristics of the inflow to the BHV, and (iii) depends on the individual leaflet. In the computational fluid-structure interaction (FSI) study, conducted for the same BHV at a cardiac output of 5 l/min, we aimed to investigate the associated flow structures. For the T-mode, we observed flow separation during the traveling wave motion, resulting in large-scale vortex shedding at the end of each T-mode cycle. For the V-mode, we saw small-scale vortex shedding superimposed with the larger vortices due to T-mode LF.

Similar to the observation that LF is more likely for higher flow rates (Figure 5), the fluttering frequency, amplitude and the number of waves also increase with CO (Figure 6), as reported also by Avelar et al. [23]. This suggests that leaflet fluttering is a fluid-structure interaction phenomenon governed by the Reynolds number of the flow through the valve [12, 13, 23]. The inflow toward the BHV was also found to affect LF. The EXP insert smoothly accelerates the flow in the left ventricular chamber of the pulsatile flow loop, thereby reducing the level of inflow disturbances to the BHV. The EXP insert exhibited less LF (Figure 5A) and lower amplitudes (Figure 6) compared to NO insert. This suggests that LF might be enhanced with higher levels of inflow disturbances.

LF was found to be leaflet-dependent, with T-mode frequently found on leaflet 1, but rarely on leaflet 3, where the V-mode dominated (Figure 5). Asymmetries in the flow loop can be excluded as a cause for these observations, because different orientations of the valve within the test cell did not significantly affect the results. In contrast, numerical results showed both T-mode and V-mode for each leaflet (Figure 7A). However, one leaflet seemed to move slightly out of phase and with a lower amplitude of the T-mode compared to the other two leaflets. Upon visual inspection of the (experimental) BHV, no apparent asymmetries or imperfections were found. Therefore, the results of the current study suggest that leaflet fluttering is sensitive to very small variations in the mechanical and geometrical properties of the BHV and leaflet. This is in agreement with the findings of Peacock [10], Thubrikar et al. [18], and Avelar et al. [21]. Asymmetric fluttering was also reported in the computational study of Corso and Obrist [22].

The continuous V-mode fluttering was found to have a frequency that increases with higher CO (Figure 6A), but this increase is independent of the inflow (Figure 6E). This might indicate that V-mode fluttering is an instability process that scales with the flow velocity. Additionally, the EXP insert could eliminate the V-mode (Figure 5A) for lower CO. This may indicate that V-mode is a self-sustained oscillation with a proper eigenfrequency that can be triggered by flow disturbances or a sufficiently high Reynolds number (i.e., elevated CO). The numerical study shows that the V-mode is associated with small-scale vortex shedding from the leaflet tips (Figure 7B, C).

In contrast to V-mode fluttering, T-mode waves appeared as single events. The number of waves and the amplitude increased with higher CO (Figure 6B, D). The numerical study indicates that T-mode is caused by vortex buildup and shedding at the base of the leaflet belly. This suggests that a smaller leaflet belly could reduce T-mode LF. Similar to the V-mode, the leaflets were not equally affected by the T-mode, which could also be attributed to slight differences in leaflet properties. T-mode waves were observed during mid and late systole (also reported by Moore and Dasi [13]). During early systole, the acceleration of the fluid may stabilize the flow through the BHV orifice, causing an increase in fluid pressure within the valve, which also stabilizes the leaflets in their open position. During mid-to-late systole, these two effects are weakened or even reversed (during flow deceleration), such that flow disturbances increase, and leaflets are destabilized. Trains of multiple T-mode waves (at most 7 waves in our experiments) could also be interpreted as vibrations with frequencies of 30-90 Hz which is in good agreement with the computational results (67 Hz). Most likely, these wave trains can be associated with high-amplitude, low-frequency fluttering reported in several other studies [12, 13, 15], and with fluttering observed *in vivo* using continuous-wave Doppler [10, 11]. However, longer trains of T-mode waves were infrequent in the current study, and interpreting these waves as single events appears more plausible. Finally, the T-mode waves observed in the present study align well with the LF reported in the computational studies by Corso and Obrist [22] and Bornemann and Obrist [25, 26].

### Limitations

The experimental and computational studies are limited by the use of only one bioprosthetic valve design and size. It is expected that different valve designs will affect LF in terms of frequency, occurrence, and amplitude. Another limitation is that the flow loop was neither designed to mimic the complex flow pattern within the left ventricle, nor to provide access to the resulting inflow profile to the valve. However, the impact of the inflow profile was investigated by comparing LF with the NO inflow insert to the case with the EXP inflow insert which is expected to reduce inflow disturbances. To further investigate whether LF was caused by disturbances in the inflow profile, we performed an FSI study in which the applied fringe region technique was used to minimize inflow disturbances by damping transverse velocity components, thereby creating a symmetrical inflow profile. The results of the FSI study imply that fluttering is an FSI phenomenon and an instability process, and that inflow disturbances are not the main drivers of the LF. This phenomenon has also been observed in 2D and 3D in our previous studies [25, 26]. Further, the aortic root model featured no coronary arteries, which is expected to impact the flow patterns in the sinus portions behind the leaflets. Because coronary flow is smallest during systole, we believe this has only modest effect on LF. Finally, the FSI study did not explore the sensitivity of LF to fluid viscosity, pulse waveform and material parameters.

### Clinical Impact

The clinical relevance of leaflet fluttering remains unclear, although several authors have suggested that leaflet fluttering may contribute to the limited durability of BHVs. Clinically, the resolution of today’s imaging modalities is not sufficient to identify V-mode fluttering *in vivo*. However, T-mode waves could potentially provide relevant diagnostic information on structural changes (e.g., leaflet thickening) or obstructions in the LVOT leading to flow disturbances. Despite these limitations in clinical assessment of leaflet fluttering, it should be emphasized that both modes have the potential to be critical factors decreasing valve durability.

## Conclusion

In this systematic and quantitative study of a specific BHV, two different modes of leaflet fluttering were identified: V-mode is a high-frequency, low-amplitude vibration of the leaflet tip, and T-mode is an intermittent, large-amplitude wave travelling from leaflet base to leaflet tip. The occurrence, amplitude, and frequency of the fluttering modes were found to scale with the CO and to depend on the individual leaflet. Our observation highlights the complexity of leaflet fluttering, which appears to be influenced by global hemodynamic parameters, local hydrodynamic instabilities, and the mechanical properties of the leaflets.

## Supplementary Information

Below is the link to the electronic supplementary material.

Supplementary file1 (MP4 37931 kb)

Supplementary file2 (PDF 126 kb)

Supplementary file3 (PDF 119 kb)

Supplementary file4 (PDF 468 kb)

Supplementary file5 (PDF 805 kb)

Supplementary file6 (XLSX 19 kb)
