# Supplementary figures and images for: Modes of Leaflet Fluttering: Quantitative Characterization of a Bovine Bioprosthetic Heart Valve
Source: Ann Biomed Eng. 2025 Nov 14;54(2):410–21. doi: 10.1007/s10439-025-03906-9 (PMC12852234; doi:10.1007/s10439-025-03906-9)

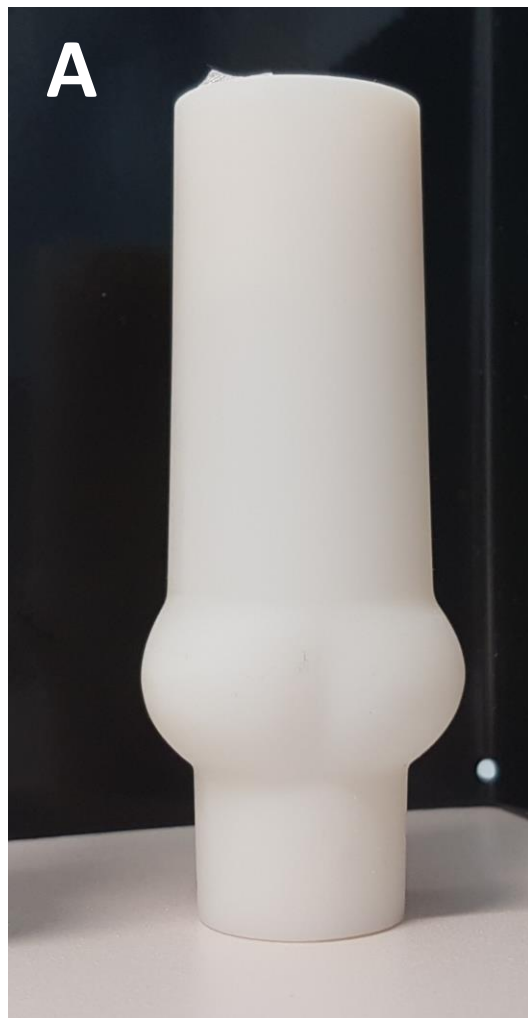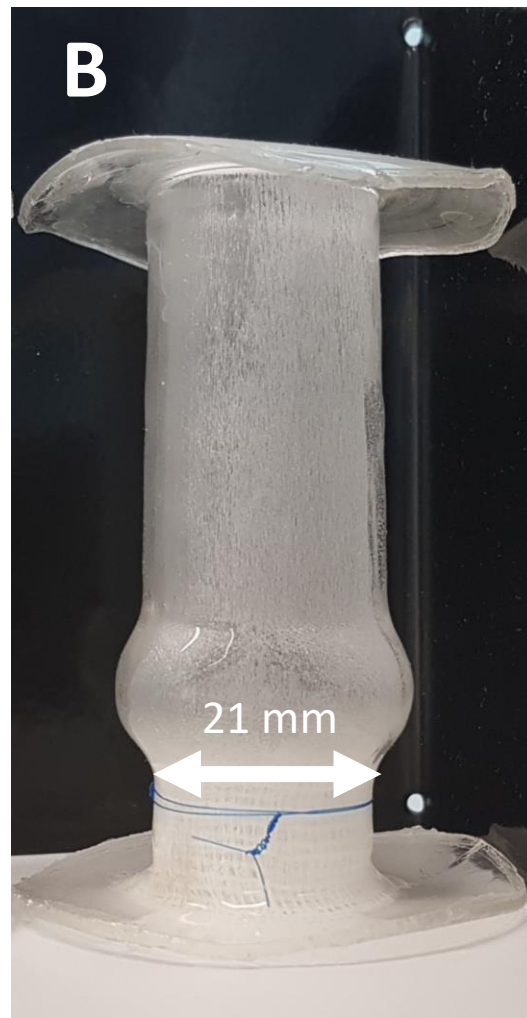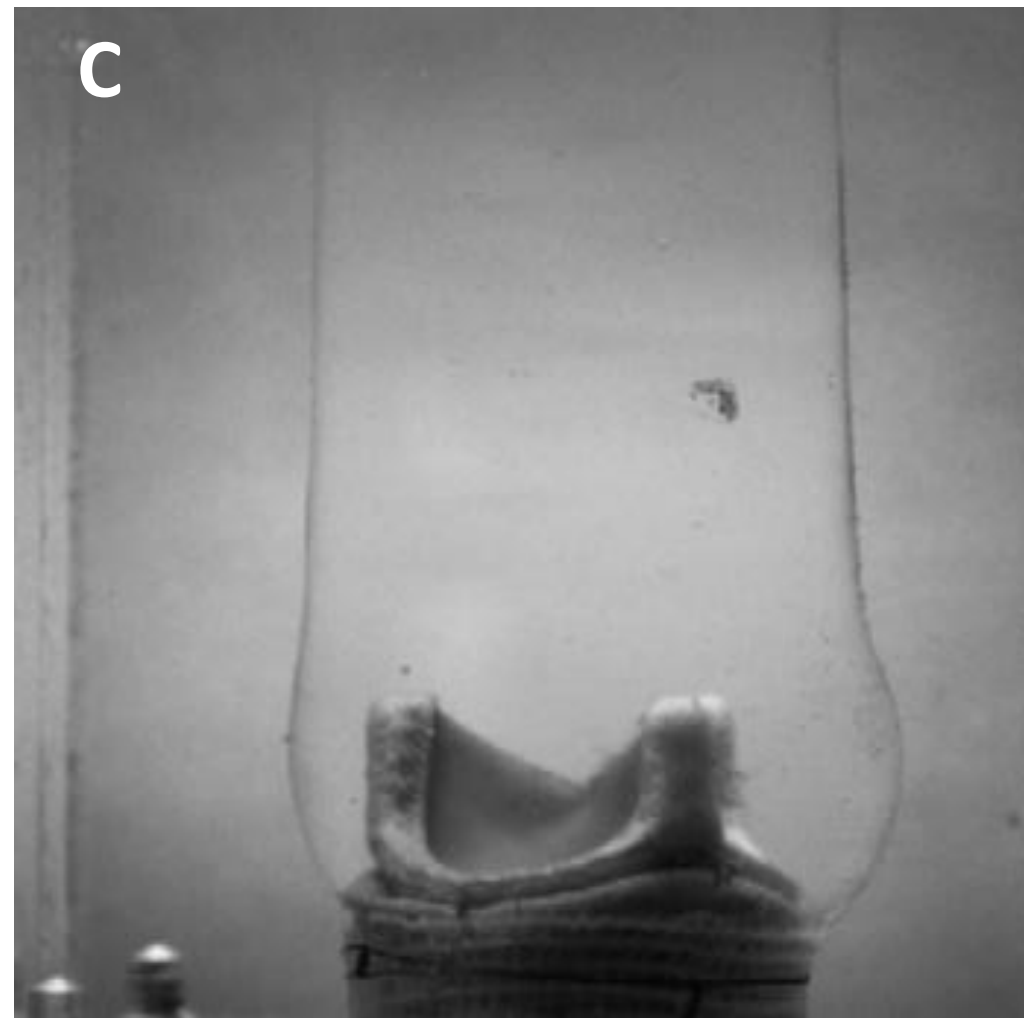

Supplement: Supplementary file 2 — Supplementary file2 (PDF 126 kb) [file 10439_2025_3906_MOESM2_ESM.pdf]

# Exponential inflow insert

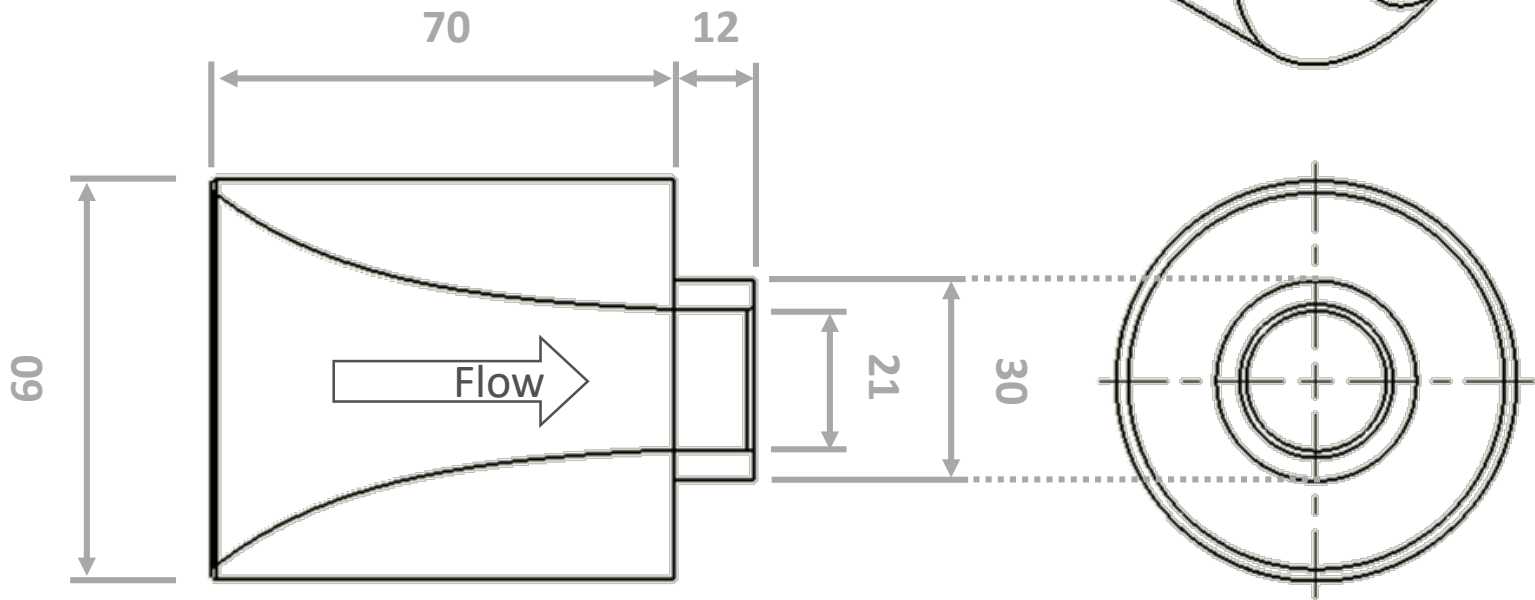

Units: mm

Supplement: Supplementary file 3 — Supplementary file3 (PDF 119 kb) [file 10439_2025_3906_MOESM3_ESM.pdf]

CO 3.5 l/min

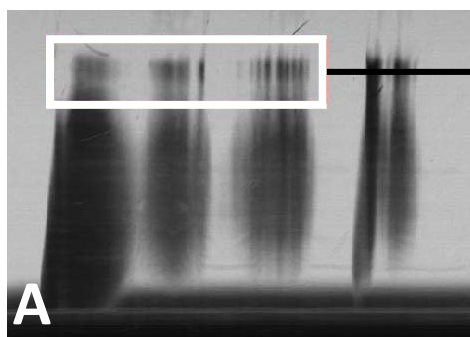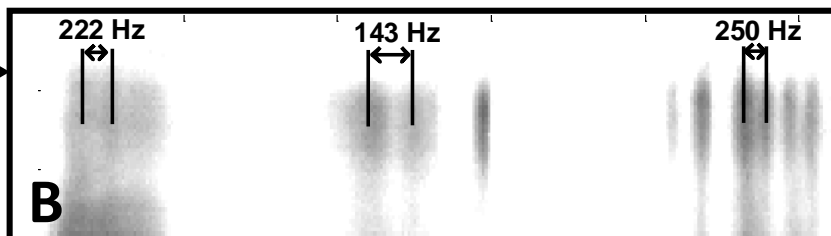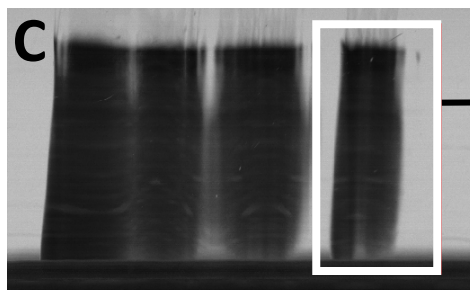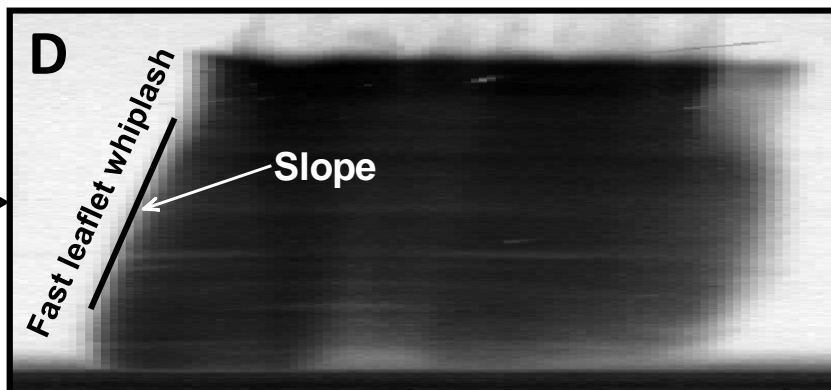

CO 5.0 l/min

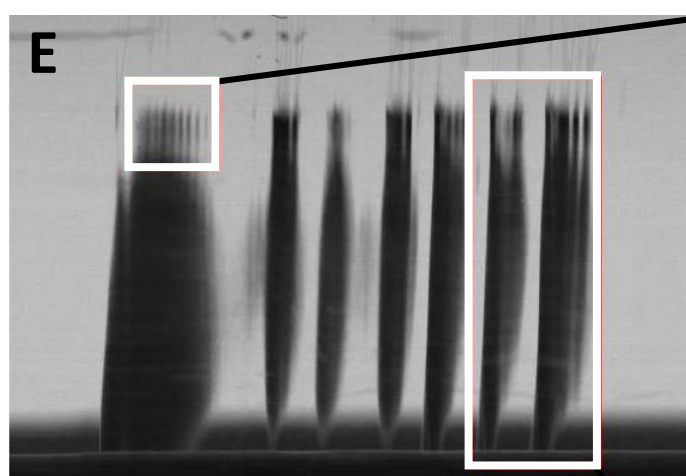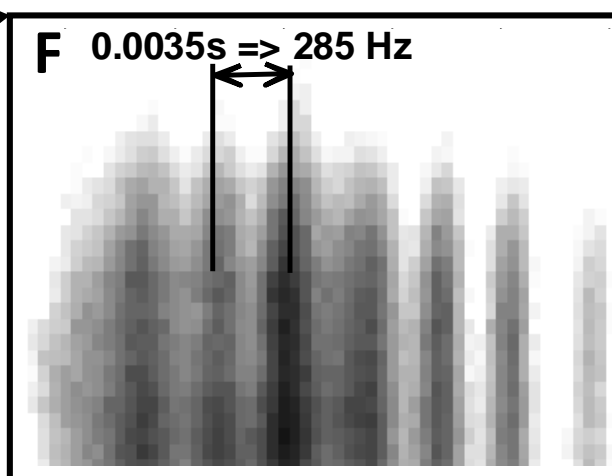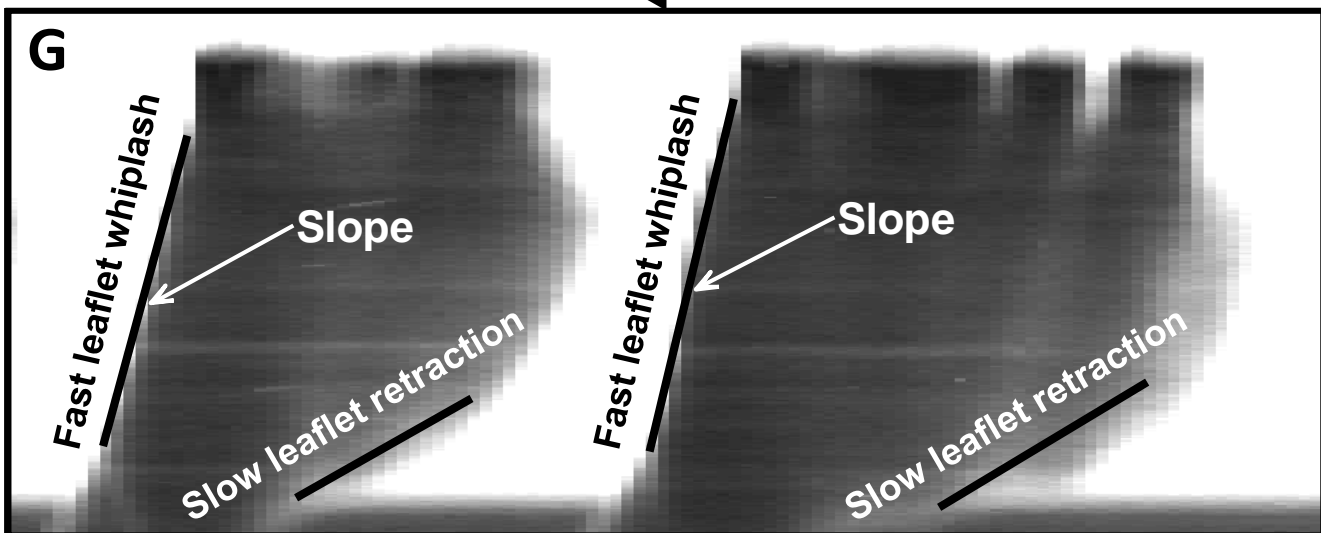

Supplement: Supplementary file 4 — Supplementary file4 (PDF 468 kb) [file 10439_2025_3906_MOESM4_ESM.pdf]
