# Supplementary material for: Modes of Leaflet Fluttering: Quantitative Characterization of a Bovine Bioprosthetic Heart Valve
Source: Ann Biomed Eng. 2025 Nov 14;54(2):410–21. doi: 10.1007/s10439-025-03906-9 (PMC12852234; doi:10.1007/s10439-025-03906-9)
Supplement: Supplementary file 5 — Supplementary file5 (PDF 805 kb) [file 10439_2025_3906_MOESM5_ESM.pdf]

Supplementary materials:

Modes of leaflet fluttering: quantitative characterization of a bovine bioprosthetic heart valve

Journal: Annals of Biomedical Engineering

Silje Ekroll Jahren[1]**, Bernhard Vennemann[1, 2]**, Karoline-Marie Bornemann[1,3], Thomas Rösgen[2], Dominik Obrist[1]

** shared first authorship

[1]ARTORG Center for Biomedical Engineering Research, University of Bern, Bern, Switzerland

[2]Institute of Fluid Dynamics, ETH Zürich, Zürich, Switzerland

[3]Department of Pediatrics (Cardiology), Stanford University, Stanford, CA, USA

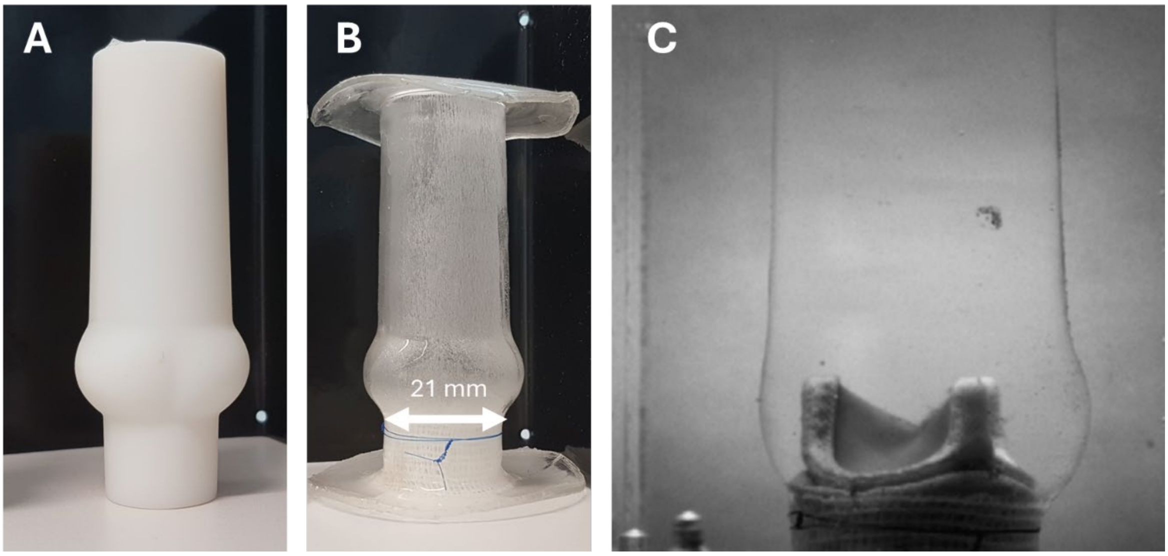

Supplementary Figure 1: Aortic root phantom fabrication: A) negative core of the phantom geometry to which silicone was added layer by layer, B) finished silicone phantom and C) phantom mounted in flow loop and with implanted bioprosthetic valve..

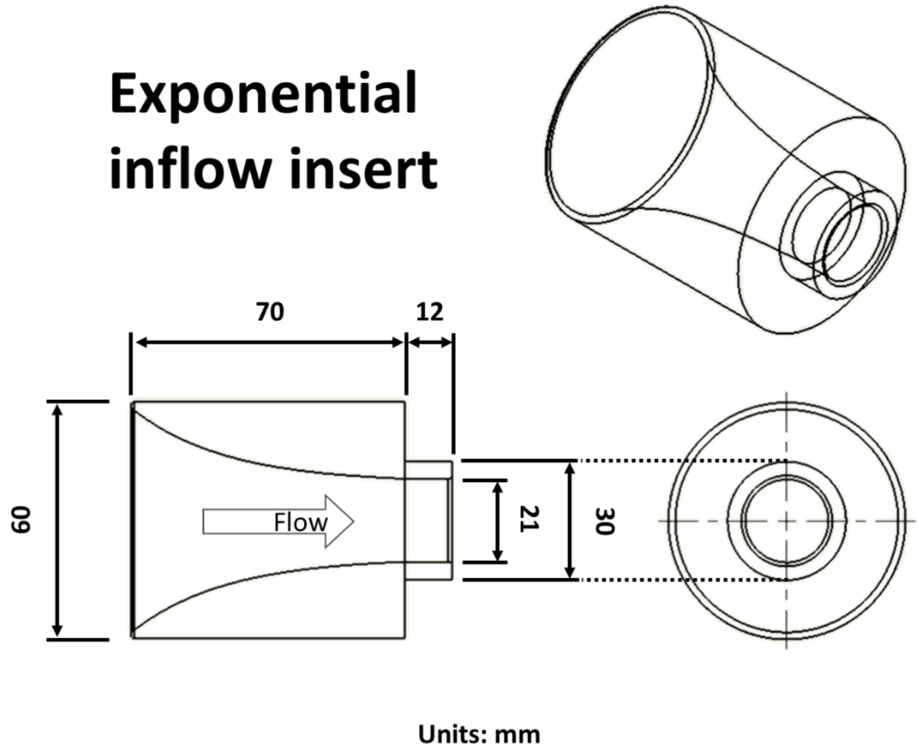

Supplementary Figure 2: Technical drawing of the exponential (EXP) inflow insert with dimensions in mm.

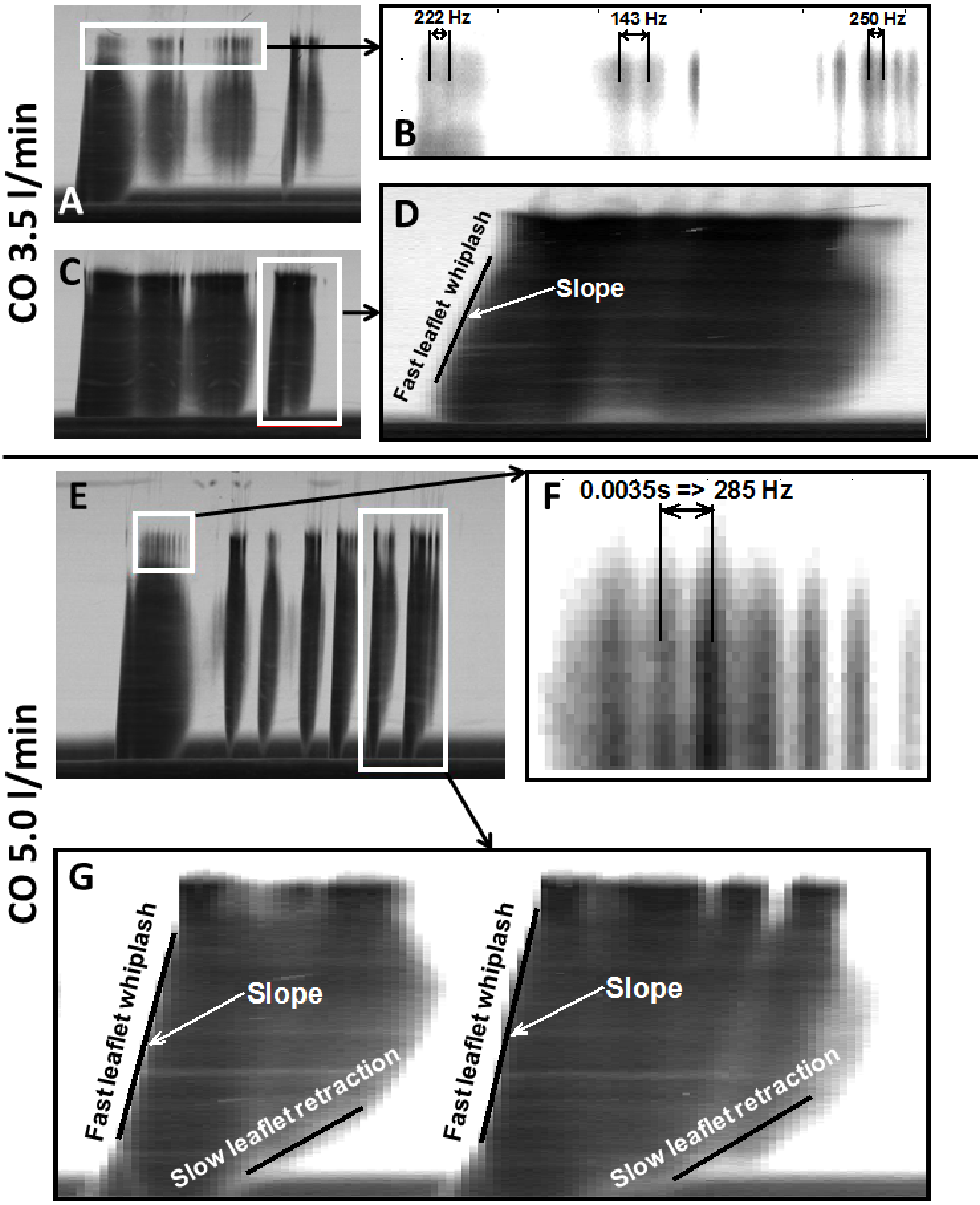

Supplementary Figure 3: A), B) High frequency tip fluttering (V-mode) and C), D) slow travelling waves (T-mode). E), F) V-mode and G) T-mode fluttering. The slopes in D and G indicate the wave velocities of the T-mode waves.

Supplementary Table 1: Overview of all measurements performed including resulting leaflet fluttering modes (T and V), frequency, amplitude and occurrence.

Inflow insert [-]	Hemodynamic condition [-]	Measurement nr. [-]	leaflet nr. [-]	Leaflet rotational orientation [-]	sinus nr. [-]	Fluttering mode [-]	Maximum frequency V-mode [Hz]	Maximum amplitude V-mode [mm]	Number of T-mode waves [-]	Maximum amplitude T-mode [mm]	T-mode frequency [Hz]
NO	1	1	1	bottom	1	T			1	1.2	
			2	right	2	V	200	0.4	0		
			3	left	3	V	200	0.3	0		
		2	1	left	1	T			1	1.19	
			2	bottom	2				0		
			3	right	3	V	210	0.3	0		
		3	1	right	1	T			2	1	15-20
			2	left	2	V	150	0.2	0		
			3	bottom	3	V	200	0.4	0		
	2	4	1	bottom	1	V,T	285	0.4	2	2.01	25
			2	right	2	V,T	260	0.5	5	2.17	50-60
			3	left	3	V	285	0.6	0		
		5	1	left	1	V,T	285	0.4	5	1.6	30-40, 90
			2	bottom	2	V,T	180	0.4	6	2.29	50-60
			3	right	3	V	290	0.6	0		
		6	1	right	1	V,T	170	0.3	2	1.21	15-20
			2	left	2	V,T	310	0.3	7	1.21	60
			3	bottom	3	V	330	0.5	0		
EXP	1	7	1	bottom	1	T			1	1.32	
			2	right	2				0		
			3	left	3	V	220	0.4	0		
		8	1	left	1				0		
			2	bottom	2				0		
			3	right	3	V	200	0.3	0		
		9	1	right	1				0		
			2	left	2				0		
			3	bottom	3	V	210	0.3	0		
		10	1	left	3	T			2	1.95	30
			2	bottom	1				0		
			3	right	2	V	210	0.2	0		
		11	1	right	2	T			3	1.97	
			2	left	3				0		
			3	bottom	1	V	210	0.2	0		
	2	12	1	bottom	1	T			3	1.17	90
			2	right	2	V,T	290	0.2	2	0.93	85
			3	left	3	V	360	0.2	0		
		13	1	left	1	V	280	0.2	0		
			2	bottom	2	V,T	200	0.3	1	0.78	
			3	right	3	V	360	0.3	0		
		14	1	right	1	V	285	0.2	0		
			2	left	2	V,T	280	0.2	6	0.91	85
			3	bottom	3	V	380	0.2	0		
		15	1	left	3	T			4	2.58	85
			2	bottom	1	V,T	210	0.2	1	0.3	
			3	right	2	V	350	0.3	0		
		16	1	right	2	T			2	2.49	25
			2	left	3	V,T	260	0.3	1	0.8	
			3	bottom	1	V	370	0.2	0		
